# One-stop-shop cardiac CT: 3D fusion of CT coronary anatomy and myocardial perfusion for guiding revascularization in complex multivessel disease

**DOI:** 10.1007/s12350-015-0324-z

**Published:** 2015-12-02

**Authors:** Alexander R. van Rosendael, Aukelien C. Dimitriu-Leen, José M. Montero-Cabezas, Jeroen J. Bax, Lucia J. Kroft, Arthur J. H. A. Scholte

**Affiliations:** 1 Department of Cardiology, Leiden University Medical Center, Leiden, The Netherlands; 2 The Interuniversity Cardiology Institute of the Netherlands, Utrecht, The Netherlands; 3 Department of Radiology, Leiden University Medical Center, Leiden, The Netherlands

## Case

**Figure 1:**
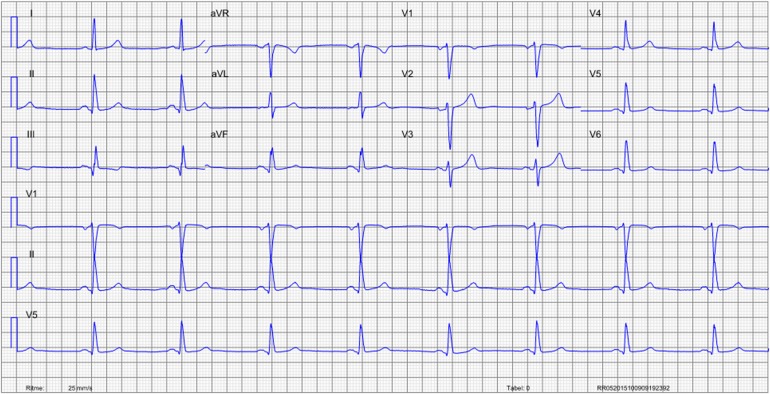
Patient’s 12-lead electrocardiogram showing sinus rhythm with Q-waves in leads II, III, and aVF, indicating an old myocardial infarction in the inferior wall

**Figure 2:**
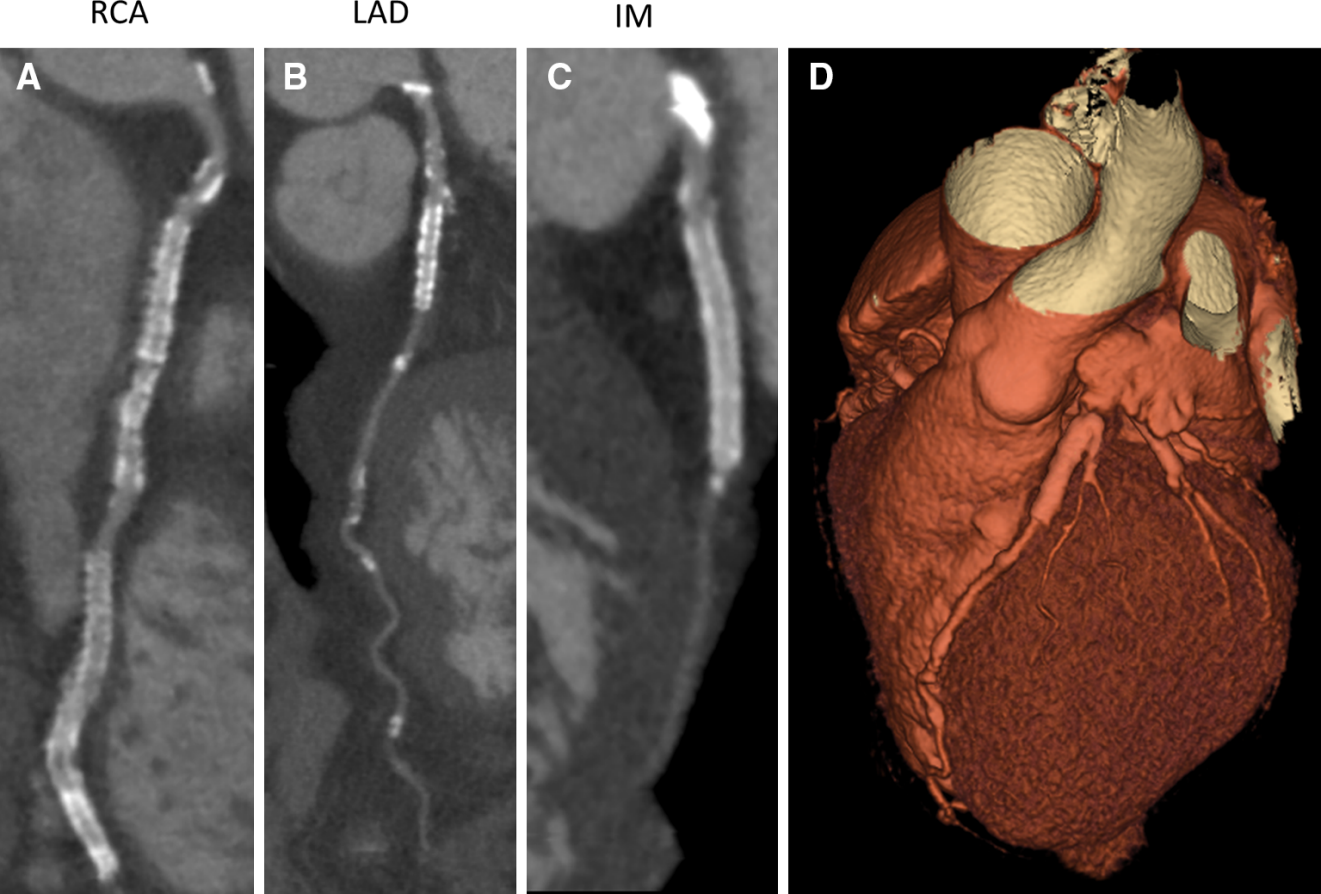
Coronary CTA depicting 2 stents in the RCA (**A**), 1 stent in the proximal LAD (**B**), and 1 stent in the IM (**C**). (**D**) 3D model of the heart and coronary arteries. *CTA*, coronary computed tomography angiography; *RCA*, right coronary artery; *LAD*, left ascending coronary artery; *IM*, intermediate branch

**Figure 3:**
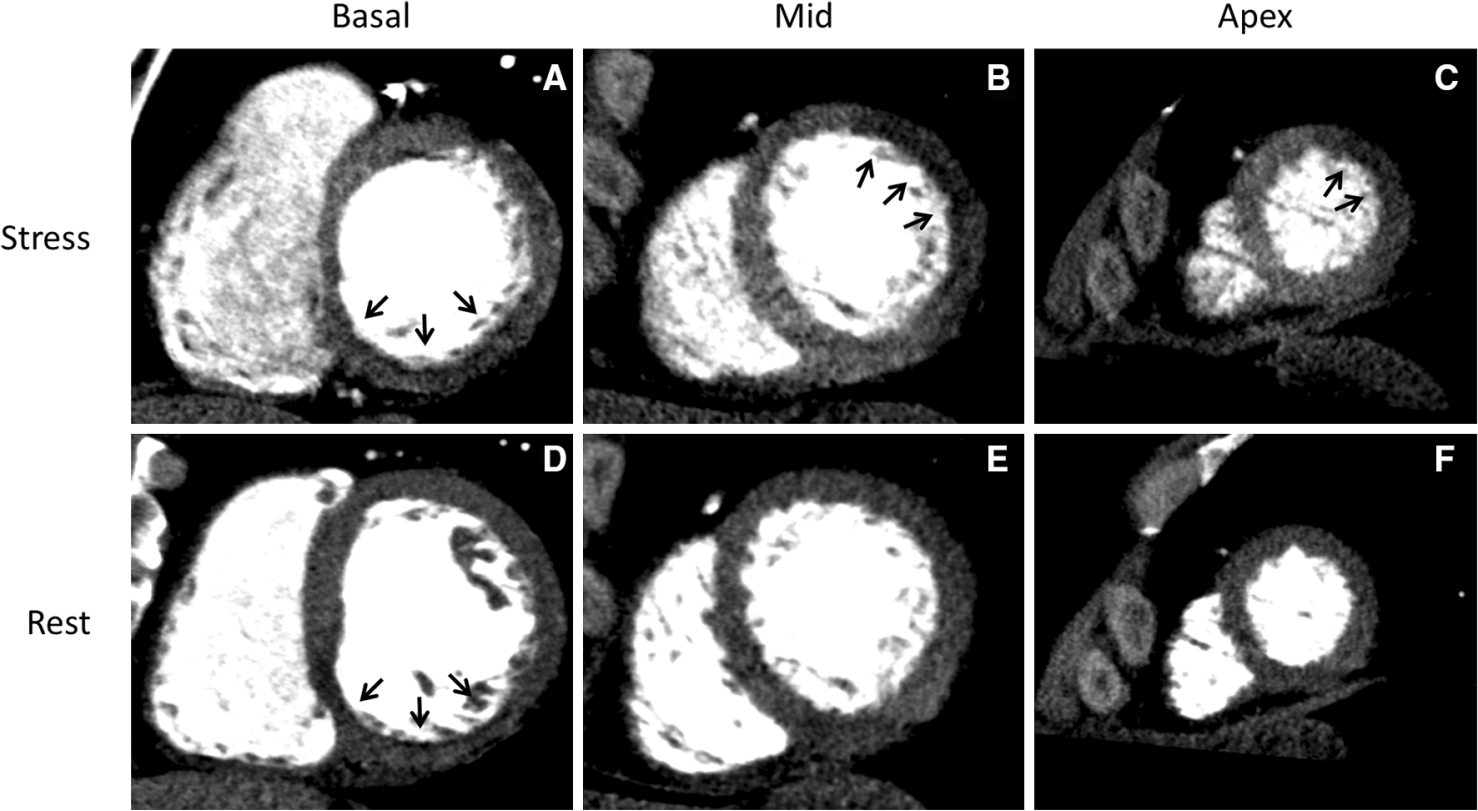
Short-axis views of CTP images at stress and rest. (**A**, **D**) At the basal level, hypo-enhancement in the inferior wall (*arrows*) is depicted at rest and stress indicating prior myocardial infarction. (**B**, **C**) At stress, hypo-enhancement in the anterolateral wall (*arrows*) at mid and apical level. (**E**, **F**) Normal myocardial enhancement at rest. *CTP*, myocardial computed tomography perfusion

**Figure 4:**
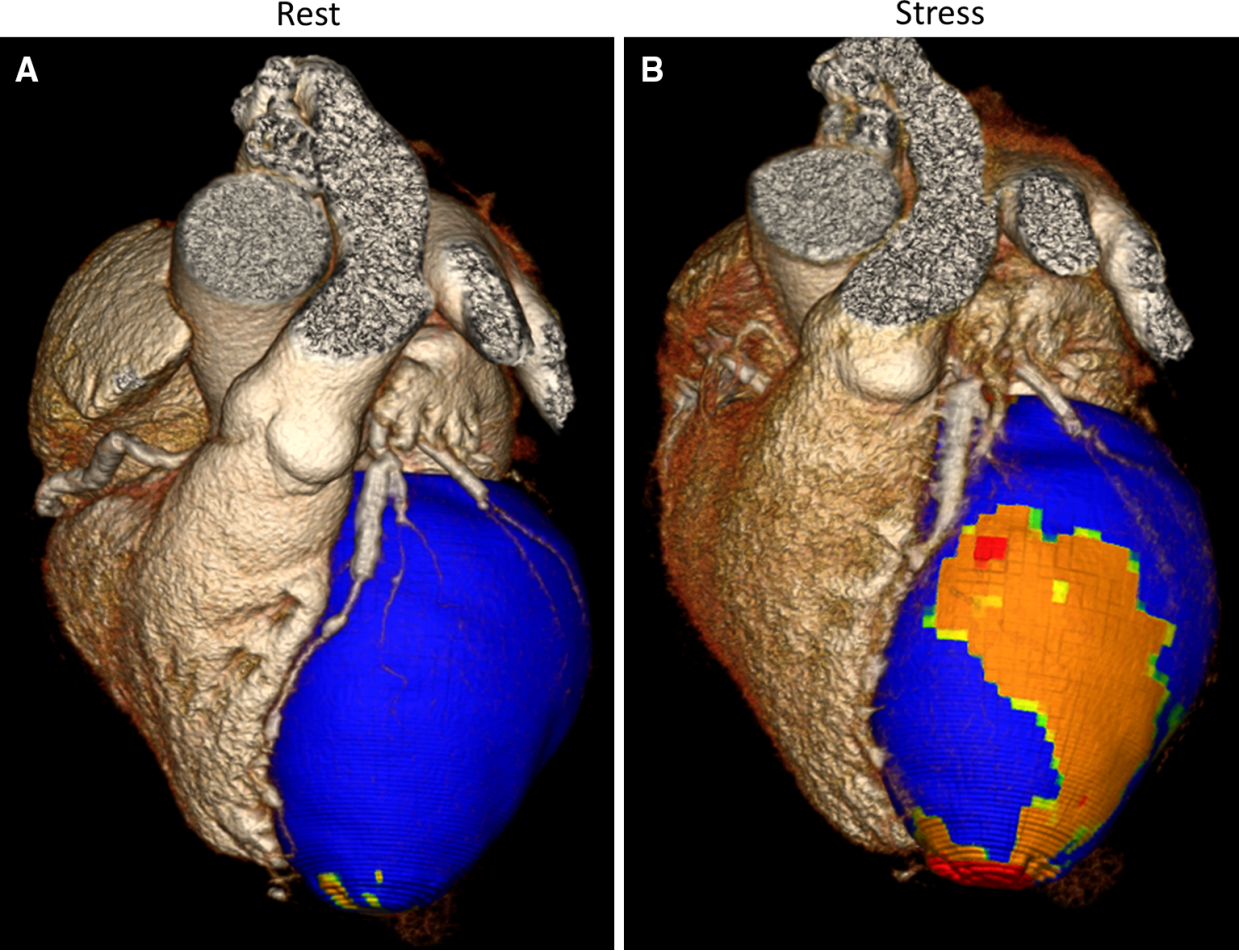
3D fusion of coronary CTA and CTP demonstrating normal myocardial enhancement at rest (**A**) and myocardial ischemia downstream the D1 at stress (**B**). *CTA*, coronary computed tomography angiography; *CTP*, myocardial computed tomography perfusion; *D1*, first diagonal branch

**Figure 5:**
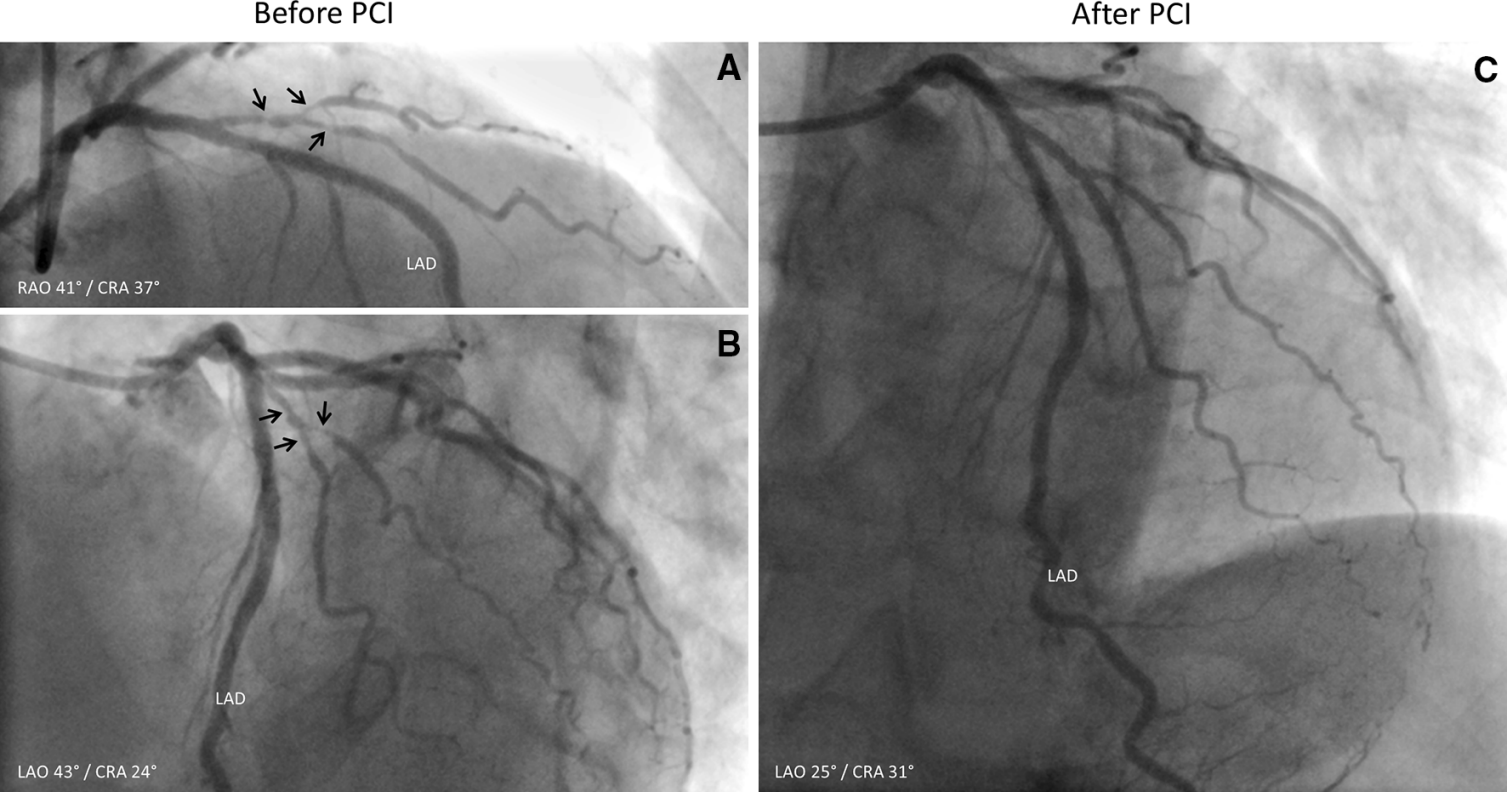
Invasive coronary angiography depicting significant lesions in a large D1 (*arrows*) (**A**, **B**) which were revascularized (**C**). *PCI,* percutaneous coronary intervention; *LAD*, left anterior descending artery; *D1*, first diagonal branch

A 59-year old male, with a history of inferoposterior myocardial infarction and multiple coronary stenting, presented to the out-patient clinic with exercise-related chest discomfort. The electrocardiogram showed sinus rhythm with Q-waves in the inferior leads (Figure 1) . Coronary computed tomography angiography (CTA) showed stents in the right coronary artery (RCA), left anterior descending artery (LAD), and intermediate branch (IM), however, obstructive coronary artery disease (CAD) could not be reliably assessed (Figure 2). Sequentially, adenosine stress CT myocardial perfusion (CTP) was performed and indicated anterolateral ischemia and the old inferoposterior scar (Figure 3). Using 3-Dimensional (3D) fusion of the coronary anatomy and stress perfusion images, the new myocardial ischemia could be allocated to the territory of the first diagonal branch (D1) (Figure 4). Invasive coronary angiography demonstrated patency of the previous stents and a significant lesion of the proximal D1, which was successfully stented (Figure 5).

## Discussion

To perform CT coronary anatomy and myocardial perfusion imaging in the same setting is an efficient way to diagnose an old myocardial infarction, new myocardial ischemia, and to allocate the ischemia to its corresponding coronary artery by 3D fusion and thereby guiding the revascularization procedure; the so-called one-stop-shop!

